# How to test for the red reflex in a child

**Published:** 2014

Examination of pupil reflections, also known as the red reflex text, can reveal problems in the cornea, lens and sometimes the vitreous, and is particularly useful in young children. These photographs show what can occur in the case of certain major eye conditions, the most serious of which is retinoblastoma.

It is essential to test the red reflex after birth, at the age of six weeks and also during routine consultations or when parents are concerned about the child's vision or the appearance of her or his eyes.

The test can alert us to large lesions in the retina. It cannot be used, however, to identify causes of poor vision related to retinal or optic nerve damage, such as retinal dystrophy or optic atrophy. For this, appropriate referral is needed.

**Note:** A dim red reflex may reveal an unequal or high refractive error.

## Procedure

- The red reflex is much easier to see in a darkened room, so switch off the lights, draw the curtains or ask the parents and child to accompany you into a room which does not have a window.
- Use a direct ophthalmoscope with the lens power set at ‘0’. Make sure the batteries are charged.
- Sit about half a metre (50 cm) away. Hold the ophthalmoscope close to your eyes.
- Encourage the child to look at the light source and direct the light at the child's eyes individually and together. You should see an equal and bright red reflex from each pupil.
- Pay attention to the colour and brightness of the red reflex. It should be identical in both eyes (Figure 1). Any difference between the eyes, an absence of the red reflex or an abnormal colour (Figures 2–4) may indicate a serious illness.

NOTE: The precise colour of the red reflex will depend on the degree of pigmentation in the eye. To determine whether the red reflex is normal, comparison with the red reflex of a parent of the child maybe helpful. If you are not sure whether the reflex is normal, dilate the pupil for a complete examination.

**Figure:**
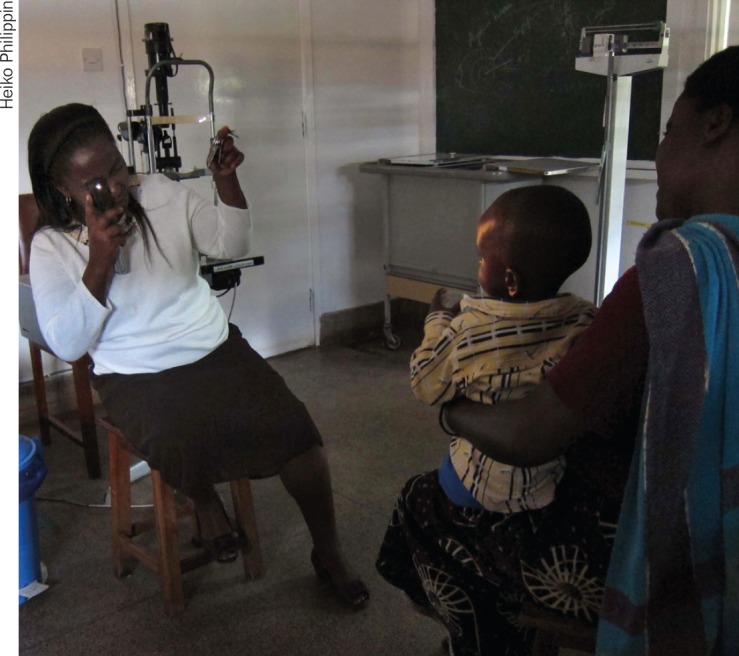
The red reflex is easier to see in a darkened room

**Figure:**
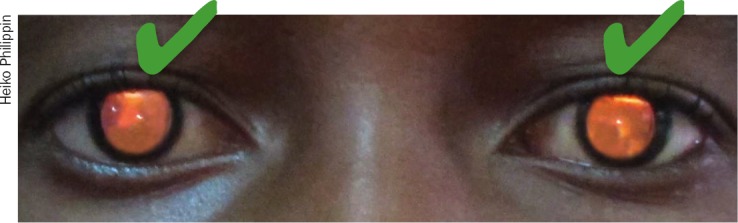
Figure 1. The normal red reflex

**Figure:**
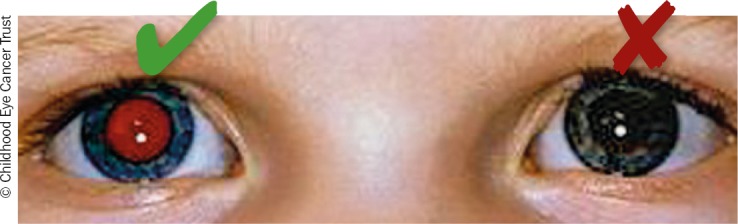
Figure 2. Right eye: the normal red reflex. Left eye: the absence of a red reflex is abnormal and could indicate a serious condition. Refer the child to a specialist

**Figure:**
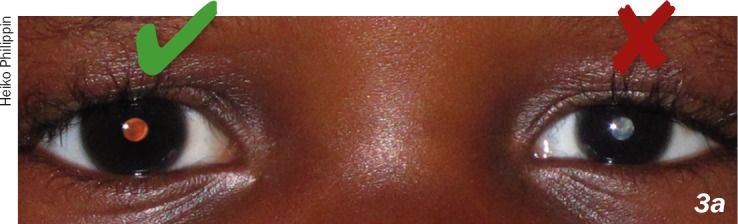
Figure 3. The wrong colour in a red reflex is abnormal and could indicate a serious condition 3a. The child in this image has a cataract in the left eye. Refer the child to a specialist

**Figure:**
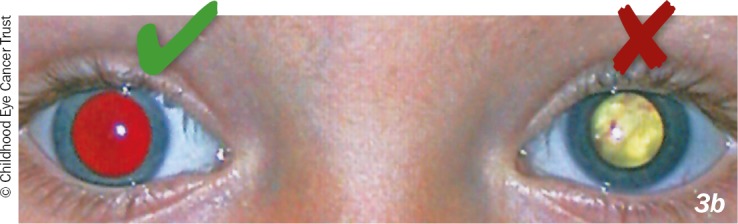
3b. The child in this image may have retinoblastoma in the left eye. Refer the child to a specialist

**Figure:**
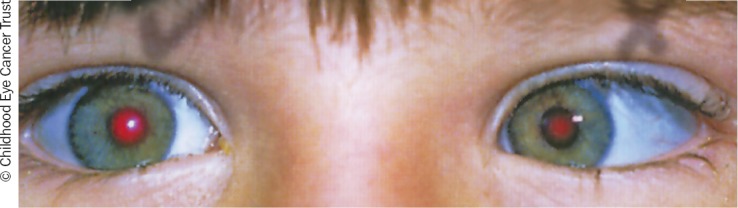
Figure 4. The red reflex is less bright in the left eye and the corneal reflection is not centred. This is a squint, which maybe the result of a serious underlying condition. Even if there is no underlying condition, squint may lead to amblyopia (loss of function in the visual cortex), which is irreversible if not treated urgently. Refer the child to a specialist


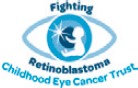
 Adapted from the poster: ‘See RED’ produced by JR Ainsworth, UK National Retinoblastoma Service, Birmingham, UK and the Childhood Eye Cancer Trust. www.chect.org.uk First published in the *Community Eye Health Journal* French edition, Issue 8, 2011.

